# Revised recommendations for health monitoring of non-human primate colonies (2018): FELASA Working Group Report

**DOI:** 10.1177/0023677219844541

**Published:** 2019-05-08

**Authors:** Ivan Balansard, Lorna Cleverley, Keith L Cutler, Mats G Spångberg, Kevin Thibault-Duprey, Jan AM Langermans

**Affiliations:** 1 Centre d’Exploration Fonctionnelle et de Formation, Campus Médecine Santé, Marseille, France; 2 Marshall Bioresources, Hull, UK; 3 Endell Veterinary Group, Salisbury, UK; 4 Astrid Fagræus Laboratory, Karolinska Institute, Sweden; 5 Animal Models & Bioimaging, Sanofi Pasteur R&D, France; 6 Animal Science Department, Biomedical Primate Research Centre, The Netherlands; 7 Department of Animals in Science & Society, Faculty of Veterinary Medicine, Utrecht University, The Netherlands

**Keywords:** husbandry, care, laboratory animal welfare, quality assurance/control

## Abstract

The genetic and biological similarity between non-human primates and humans has ensured the continued use of primates in biomedical research where other species cannot be used. Health-monitoring programmes for non-human primates provide an approach to monitor and control both endemic and incoming agents that may cause zoonotic and anthroponotic disease or interfere with research outcomes. In 1999 FELASA recommendations were published which aimed to provide a harmonized approach to health monitoring programmes for non-human primates. Scientific and technological progress, understanding of non-human primates and evolving microbiology has necessitated a review and replacement of the current recommendations.

These new recommendations are aimed at users and breeders of the commonly used non-human primates; *Macaca mulatta* (Rhesus macaque) and *Macaca fascicularis* (Cynomolgus macaque). In addition, other species including *Callithrix jacchus* (Common marmoset) *Saimiri sciureus* (Squirrel monkey) and others are included. The important and challenging aspects of non-human primate health-monitoring programmes are discussed, including management protocols to maintain and improve health status, health screening strategies and procedures, health reporting and certification. In addition, information is provided on specific micro-organisms and the recommended frequency of testing.

## Résumé

La similarité génétique et biologique entre les primates non humains et les humains a assuré le maintien de l'utilisation des primates dans la recherche biomédicale où d'autres espèces ne peuvent pas être utilisées. Les programmes de surveillance sanitaire des primates non humains fournissent une approche visant à surveiller et à contrôler les agents endémiques et entrants susceptibles de provoquer des maladies zoonotiques et anthroponotiques ou d’interférer avec les résultats de recherche. Les recommandations FELASA ont été publiées en 1999. Elles visaient à mettre en place une approche harmonisée des programmes de surveillance sanitaire des primates non humains. Les progrès scientifiques et technologiques, la compréhension des primates non humains et l'évolution de la microbiologie ont rendu nécessaire d’examiner et de remplacer les recommandations actuelles. Ces nouvelles recommandations s'adressent aux utilisateurs et aux éleveurs de primates non humains communément utilisés ; à savoir le macaque rhésus (*Macaca mulatta*) et le macaque cynomolgus (*Macaca fascicularis*). D'autres espèces sont en outre incluses, dont le *Callithrix jacchus* (ouistiti commun), le *Saimiri sciureus* (singe écureuil) et d'autres. Les aspects importants et difficiles des programmes de surveillance sanitaire des primates non humains sont abordés, notamment les protocoles de gestion pour maintenir et améliorer l'état de santé, les stratégies et procédures de dépistage, les rapports de santé et la certification. Des informations sur les micro-organismes spécifiques et la fréquence des analyses recommandées sont également fournies.

## Abstract (de)

Die genetische und biologische Ähnlichkeit zwischen nicht-menschlichen Primaten und dem Menschen hat für eine kontinuierliche Verwendung von Primaten in der biomedizinischen Forschung gesorgt, wenn andere Arten nicht verwendet werden können. Gesundheitsmonitoring-Programme für nicht-menschliche Primaten bieten einen Ansatz zur Überwachung und Kontrolle sowohl endemischer als auch eingeführter Erreger, die Zoonosen und Anthroponosen verursachen oder Forschungsergebnisse beeinträchtigen können. 1999 wurden FELASA-Empfehlungen veröffentlicht, die einen harmonisierten Ansatz für Gesundheitsmonitoring-Programme für nicht-menschliche Primaten bezweckten. Der wissenschaftlich-technologische Fortschritt, das Verständnis nicht-menschlicher Primaten und die sich entwickelnde Mikrobiologie haben eine Überprüfung und Aktualisierung der derzeitigen Empfehlungen erforderlich gemacht. Diese neuen Empfehlungen richten sich an Anwender und Züchter der häufig verwendeten nicht-menschlichen Primaten *Macaca mulatta* (Rhesus Macaque) und *Macaca fascicularis* (Cynomolgus Macaque). Des weiteren betreffen sie Arten wie *Callithrix jacchus* (Weißbüschelaffe), *Saimiri sciureus* (Gewöhnlicher Totenkopfaffe) und andere. Es werden wichtige und schwierige Aspekte der Gesundheitsüberwachungs-Programme für nicht-menschliche Primaten diskutiert, darunter Managementprotokolle zur Aufrechterhaltung und Verbesserung des Gesundheitszustandes, Strategien und Verfahren zum Gesundheitsscreening, Gesundheitsberichterstattung und Zertifizierung. Darüber enthalten sie Informationen über bestimmte Mikroorganismen und die empfohlene Häufigkeit von Tests.

## Resumen

La similitud genética y biológica entre los primates no humanos y los humanos ha garantizado la continuidad del uso de primates en la investigación biomédica cuando no se pueden utilizar otras especies. Los programas de control de la salud de los primates no humanos ofrecen un método para controlar y supervisar los agentes endémicos y entrantes que pueden provocar enfermedades zoonóticas y antroponóticas así como interferir en los resultados de las investigaciones. En 1999, se publicaron las recomendaciones de FELASA con la intención de presentar un método harmonizado para los programas del control de la salud de primates no humanos. El progreso científico y tecnológico, junto con el entendimiento de los primates no humanos y los avances en la microbiología han hecho que sea necesario una revisión y un reemplazo de las recomendaciones actuales. Estas nuevas recomendaciones están destinadas a usuarios y criadores de los primates no humanos más utilizados: *Macaca mulatta* y *Macaca fascicularis*. Asimismo, se incluyen otras especies como *Callithrix jacchus* y *Saimiri sciureu*, entre otras. También se debaten los aspectos importantes y complejos de los programas para el control de la salud de los primates no humanos como los protocolos de gestión para mantener y mejorar el estado de salud, procedimientos y estrategias de revisiones médicas y certificación e informes sobre salud. Finalmente, se facilita información sobre microorganismos específicos y la frecuencia recomendada para la realización de pruebas.

## Introduction

Since the publication in 1999 of recommendations for health monitoring of non-human primate colonies^1^ science has evolved. New methodologies have been developed and priorities for health screening non-human primates (NHPs) have changed with new assessments outlined in EU directive 2010/63/EU. This has necessitated a review of the current health-monitoring guidelines for NHPs.

The use of NHPs in biomedical research is still necessary for the advancement of science.^2^ This use, however, should be limited to investigations where alternative methodologies and where *in-vitro* models or other animal models are unavailable, for the preservation of the respective NHP species and in essential areas of biomedical research with a high probability of achieving benefit for human health.

The source of NHPs in biomedical research is not limited to European breeding colonies, although it is now effectively limited to purpose-bred animals. In EU member states, only NHPs that are offspring of animals that have been bred in captivity (F2/F2+ generation bred) will be permitted to be used after 10 November, 2022. Monitoring the health and improving the welfare of these animals at all stages, in both the source breeding colonies and in the research centres through a harmonized approach to husbandry and health monitoring, will ensure the suitability of NHPs in quality-driven research. In addition, since many pathogens have the ability to infect and cause disease in NHPs and also infect and cause disease in humans, this harmonized approach will also assist in reducing the risk of zoonosis to animal handlers and conversely anthroponotic infections in animals.

In contrast to many other commonly-used laboratory animal species NHPs pose considerable challenges when trying to establish and manage health status. They are complex and mostly large animals which must be co-housed in compatible social groups in an enriched environment. Gestation and time to weaning is prolonged and the reproductive rate is generally slow compared with other laboratory species. In addition, the importance of appropriate behavioural management and socialization is being increasingly recognized in the maintenance of non-human primate health and welfare, requiring the consideration of human health in addition to animal health.

All NHPs supplied for use in biomedical research should be of known health status, particularly with respect to major zoonotic pathogens such as *Cercopithecine herpesvirus 1* (Herpes simiae, Herpes B virus) and *Mycobacterium tuberculosis.*^3–5^ When supplied, they must be accompanied by a relevant lifetime veterinary history and a health declaration detailing all health screening carried out including when it was carried out, the name/details of the laboratory performing the testing and the methodology employed.

## Definitions

**Microbiological unit:** the microbiological unit is considered as the frame for health monitoring. It is possible for the microbiological unit to be a single animal but in the context of an NHP this is considered unlikely. More likely is the group of animals occupying a room, a set of rooms or a defined area which may be complicated by access to an external environment or by being serviced by the same plant or staff as other groups of animals which may link the groups in a microbiological sense. Identification of the microbiological unit is key to health monitoring and management.

**Pathogenic agent:** an infectious or parasitic agent capable of causing clinical disease and possibly mortality in immunocompetent animals.

**Opportunistic agent:** an agent which may be present within the animal’s environment or as part of the normal commensal flora without causing clinical disease but may become pathogenic in certain pathological conditions. Opportunist organisms may act synergistically with other infectious agents or in situations when immune status is compromised.

**Interfering agent:** an agent which may or may not have any pathogenic or opportunistic capacity but which has the potential to interfere with scientific aims.

**Zoonotic agent:** a pathogen which is naturally transmitted between vertebrate animals and man.

**Anthroponotic agent:** a pathogen which may be transmitted from man to animals.

## Management protocols to maintain or improve health status

The health status of any group of animals is determined by various factors interacting to exert influence, with the most significant challenge provided by exposure to infectious microorganisms. Maintaining an established health status within a colony depends on eliminating challenges from non-endemic infectious agents entering the colony and reducing the level of challenges from endemic infectious agents. The consequence of these challenges is determined by the immune status of individual animals and the exposed population as a whole. There are both direct and indirect influences exerted on the immune system associated with environmental conditions and management practices.

In order to maintain physical and mental health it is important to reduce the potential for stress or distress as these factors are known to have a negative effect on immune function. These effects can be minimized by providing an appropriate and hygienic environment together with the supply of a wholesome and balanced diet. The importance of manipulation, handling and socialization to avoid stress is also an important consideration.

Minimizing the level of challenge from endemic and non-endemic infectious agents should include separation of clinically unwell animals from the colony. Where possible, sick animals should be separated from the group for treatment to allow recovery and improve immune function.

## Endemic infections

In order to improve the health status of a colony, sources of endemic infection should be identified and action taken to reduce or prevent the transmission of the infectious agent to other non-infected animals. Maintaining the highest possible standards of hygiene and the immediate isolation of infected animals are important, but it is also important to implement appropriate health screening protocols to identify infected animals and determine appropriate action. In such situations, it is vital that realistic goals are set, and that the limitations of the available technology, including reliable animal identification and sensitivity and specificity of laboratory-testing technology are recognized and understood.

## Non-endemic infections

The greatest challenge from non-endemic infectious agents for any group of animals of any species, is likely to be as a result of the introduction of additional animals into a colony. If this is necessary, biosecurity precautions to prevent the inadvertent introduction of infectious agents must be carefully considered and implemented. This should include a detailed examination of the health status of the natal group and group of origin of the animal(s) in question and a period of quarantine. The duration of quarantine will depend on the particular pathogens of interest. During quarantine, clinical samples should be collected for appropriate laboratory testing and appropriate prophylactic or preventative medication given, or vaccination carried out as appropriate.^6^

Vaccination can be an important component of disease-control programmes to minimize the transmission of non-endemic infections in a colony, for example, some animal facilities have chosen to immunize their macaques with measles vaccine. The limitations associated with vaccination should be considered. Vaccination can, for example, reduce the prevalence or severity of a disease but may not prevent infection in animals who are unable to sero-convert. In addition, vaccination may mask an infection, resulting in the delay of treatment and promoting dissemination to unvaccinated subjects. Health-monitoring programmes can be hampered because serological analysis to determine presence or absence of disease is not easily achieved in vaccinated primates for the vaccinated agent. However, to some extent this can be overcome by using direct detection methods such as polymerase chain reaction (PCR).

A significant risk factor associated with disease transmission to NHPs is presented by wildlife, particularly rodents, insects and birds. A vector-control programme should be in place to reduce transmission risk. Animal handlers also present a significant risk as many organisms associated with human disease are also able to infect NHPs. Food may also present a risk, for example fresh fruit and vegetables can serve as vehicles of transmission.

## Health-screening strategies and protocols

Health screening at its most basic level starts with close observation of every primate by experienced staff at an appropriate frequency (at least daily). This should also include periodic clinical examination by a veterinary surgeon familiar with the species in question. Ill health often first presents as behavioural changes before clinical signs of disease become apparent. Behavioural changes including inactivity, hiding away, changes in eating or drinking, crouching and scratching excessively can be primary indicators of ill health. For example, tuberculosis in macacques may present as under-eating and weight loss without any clinical signs. Unexplained behavioural changes and clinical signs should be investigated further to establish the cause, but it must be considered that NHPs often mask signs of pain and it may be difficult to identify affected NHPs by behavioural changes.

*Post-mortem* examination of animals who die unexpectedly or are euthanized, either as a planned action or as an emergency procedure, also provide valuable information about the health status of individuals and their colony.

The most common understanding of health monitoring is the collection of samples of blood or faeces. When designing a health-screening programme the following factors should be considered:

- What sample types should be collected: blood, faeces, swabs, tissues?
- How many animals should be sampled?
- Which animals should be sampled?
- How frequently should the animals be sampled?
- Which type of analysis is appropriate: PCR, serology, culture, microscopy?

The answers to these questions depend on various factors including what is understood about the biology and epidemiology of the organism of interest.

Consideration should also be given to the aim of screening. Health screening may be used to confirm a negative colony, to determine the presence of an infectious agent or to determine the prevalence of an endemic agent. Understanding whether the host’s immune response to challenge an infection will eliminate the infectious agent without persistent, latent or chronic carrier status is also important (Table 1A–1C).

**Table 1a.** Recommended infectious agents to monitor and frequencies of monitoring for laboratory macaques.

	At arrival	At least annually
Viruses		
Rabies*, Lyssavirus (a, b)*	x	
B virus (*Herpesvirus simiae, Cercopithecine herpesvirus 1*)	x	x (b)
Filoviruses (Ebola-Reston)	x (c)	
Measles, *Morbillivirus*	x (d)	x (d’)
Retroviruses (SIV, STLV, SRV) (e)	x	
Bacteria		
*Mycobacterium*	x	x (f)
– *africanum*		
– *bovis*		
– *tuberculosis*		
*Salmonella spp.*	x	x
– *typhimurium*		
– *enteritidis*		
*Shigella spp.*	x	x
*Yersinia*	x	x
– *pseudotuberculosis*		
– *enterocolitica*		
Parasites		
*Entamoeba histolytica*	x	x
*Giardia spp.*	x	x
All Helminths including	x	x
– *Strongyloides stercoralis*		
– *Trichuris*		
– *Echinococcus multilocularis (g)*		

(a) In endemic countries, vaccination should be considered.

(b) Except for countries which are officially free from this disease and for closed colonies with NHP coming from these countries.

(c) Only for cynomolgus coming from Philippines.

(d) Except if vaccinated (d’) can be usefull in outdoor facilities (contact with humans).

(e) It may be necessary to repeat this test as a negative result is not always predictive for retroviruses.

(f) In case of closed colonies bred in Europe, and under vet supervision, TB management can be alleviated by checking less often or only on a representative sample of the population.

(g) only in outdoor facilities, in endemic areas.

Other infectious agents will be added if necessary considering the national regulation, the origin of the animals, the type of research protocol to be performed and the risk assessment.

**Table 1b.** Recommended infectious agents to monitor and frequencies of monitoring for laboratory baboons.

	At arrival	At least annually
Viruses		
Rabies, *Lyssavirus (a, b)*	x	
Retroviruses (SIV, STLV, SRV) (b, c)	x	
Bacteria		
*Mycobacterium*	x	x (d)
– *africanum*		
– *bovis*		
– *tuberculosis*		
*Salmonella spp.*	x	x
– *typhimurium*		
– *enteritidis*		
*Shigella spp.*	x	x
*Yersinia*	x	x
– *pseudotuberculosis*		
– *enterocolitica*		
Parasites		x
*Entamoeba histolytica*	x	x
*Giardia spp.*	x	x
All Helminths including	x	x
– *Strongyloides stercoralis*		
– *Trichuris*		
– *Echinococcus multilocularis (e)*		

(a) In endemic countries, vaccination could be considered.

(b) Except for animals from virus-free closed colonies.

(c) It may be necessary to renew this test as a negative result is not always predictive for retroviruses.

(d) In case of closed colonies bred in Europe, and under vet supervision, TB management can be alleviated by checking less often or only on a representative sample of the population.

(e) only in outdoor facilities, in endemic areas.

Other infectious agents will be added if necessary considering the national regulation, the origin of the animals, the type of research protocol to be performed and the risk assessment.

**Table 1c.** Recommended infectious agents to monitor and frequencies of monitoring for laboratory New World Monkeys (*Saimiri sciureus, Callithrix jacchus*).

	At arrival	At least annually
Viruses		
Rabies, *Lyssavirus (a, b)*	x	
Bacteria		
*Mycobacterium*	x	
– *africanum*		
– *bovis*		
– *tuberculosis*		
*Salmonella spp.*	x	x
– *typhimurium*		
– *enteritidis*		
*Shigella spp.*	x	x
*Yersinia spp.*	x	x
– *pseudotuberculosis*		
– *enterocolitica*		
Parasites		x
*Entamoeba histolytica*	x	x
*Giardia spp.*	x	x
All Helminths including	x	x
– *Strongyloides stercoralis*		
– *Trichuris*		
– *Echinococcus multilocularis (c)*		

(a) In endemic countries, vaccination could be considered.

(b) Except for NHP from countries which are officially free from this disease.

(c) only in outdoor facilities, in endemic areas.

Other infectious agents will be added if necessary considering the national regulation, the origin of the animals, the type of research protocol to be performed and the risk assessment.

To establish prior challenge from an infectious agent amongst a group of animals, a single positive antibody ELISA (enzyme-linked immunosorbent assay)-test result can do this, but the agent may no longer be present. If determining that a colony is negative for a particular agent, then demonstrating that every animal within the group of interest remains seronegative even on multiple occasions may not be sufficient. Seronegative latent carriers may exist in which case additional testing may be required, for example PCR.

If testing is used to determine prevalence, it may be sufficient to sample and test a proportion of the animals in the group of interest.

It is important that sampling is representative, testing appropriate animals (e.g. by age or gender) and the number of animals to be tested must be determined using valid statistical methods (Table 2).

**Table 2.** Interpretation considerations of standard screening methods used in Animal Health Monitoring.

Serology	PCR	Culture	Microscopy	Interpretation
Positive	Negative			May indicate a previous infection that has now resolved and the organism is no longer being shed.
				Can be a result of cross-reactivity of antigens used in the serology assay. Confirm assay specificity
				The sampling protocol may not be sufficient to detect organisms of low prevalence
Negative	Positive			May indicate that the infection is at a early stage prior to seroconversion.
				PCR specificity is dtermined by the specificity of the primers/probes used. Confirm specificity of the assay.
				Repeat testing after 2-3 weeks to allow seroconversion time
	Negative	Positive		A culture positive result should also give a positive PCR result as both methods rely on a direct detection.
				A delay in collecting the PCR sample may result in a sample being taken after the infection has resolved giving a negative result.
				The organism of interest may be intermittently shed and was not present in the PCR sample
				Misidentification by culture or the specificity of the primer/probe is inappropriate.
				Repeat Culture and refer for full identification to a refernce laboratory.
	Positive	Negative		PCR has greater sensitivity than culture methods and will detect positives at an earlier stage of infection.
				PCR specificity is determined by the specificity of the primers/probes used. Confirm specificity of the assay.
	Negative		Positive	The organism may be intermittently shed and was not present in the PCR sample
				Misidentification by microscopy or the specificity of the primer/probe is inappropriate.
				Repeat microscopy and refer for full identification to a refernce laboratory.
	Positive		Negative	PCR has greater sensitivity than microscopy methods and will detect positives at an earlier stage of infection.
				PCR specificity is determined by the specificity of the primers/probes used. Confirm specificity of the assay.

Understanding the epidemiology, and particularly the rate of transmission between individuals, of the infectious agent under consideration is essential. An understanding of epidemiology for each agent will also influence frequency of sampling and testing. For example, understanding that organisms may be intermittently shed is particularly pertinent when screening animals for the presence of enteric pathogens, for example, the collection of rectal swabs for culture on a single occasion or daily on three consecutive days.

Consideration should be given to the possible limitations of the laboratory-testing technology and methods employed in health screening (Table 3). Sensitivity and specificity are functions of the testing technology and define the ability of a test to truly identify status. Each of these can be altered, to some extent, usually at the expense of the other, depending on what is required to truly define status, by altering cut-off values. This may be done, for example, if there is a desire to eradicate an infectious agent from a group of animals rather than when monitoring prevalence. Predictive value, rather than being a function of the test, is a function of the prevalence of an agent within a population; a high prevalence confers a high positive predictive value (a positive result is likely to be correct) but a low prevalence confers a low positive predictive value (a positive result becomes more likely to be a ‘false’ positive as the prevalence decreases). When testing to assess prevalence, an understanding of predictive value is required to determine the number of animals that should be sampled within a population.

**Table 3.** The limitations of available test methods and recommended actions after confirming a positive result.

Organisms	Tests Available	Limitations	Purpose	Action to be Taken
Cercopithecine Herpesvirus 1, Herpesvirus simiae Herpes B	Serology	Does not detect latent Virus	Quarantine Screening	Separate positive animal from non-infected animals. Take action to protect staff from this severe zoonosis
	Mutiplex Flurorometric Immunoassay	Does not detect early disease	Annual Health Screening	
	ELISA	Assays may cross reacts with HSV-1. Confirm specificity		
	Virus isolation/Culture	Not widely available. Requires high containment 3 or 4 depending on country.	Identify genotype	
			Identify lesions	
	Polymerase Chain Reaction		Confirm negative after animal handler bite	
			Confirmation of serology positive result	
Simian Immunodeficiency Virus SIV	Serology:	Does not detect latent Virus	Quarantine Screening	Depending on the retrovirus, evaluate the risk of contamination to the colony and evaluate risk to research.
Simian T-cell Lymphotrophic Virus, STLV	Mutiplex Flurorometric Immunoassay	Does not detect early disease		
Simian type D Retroviruses, SRV(D)	ELISA	30% non-serocoversion in SRV(D)		
	Western blotting	Not widely available	Confirmation of serology positive result.	
	Polymerase Chain Reaction	SRV D RT-PCR may be serotype specific ie. *gag* genes only detect types 1-3.	Confirmation of serology positive result	
			Monitor Viral load	
Simian Foamy Viruses - SFV (Spumavirus)	Serology	Does not detect latent Virus	Quarantine Screening	Evaluate the risk to research
	Mutiplex Flurorometric Immunoassay	Does not detect early disease	Pre-screening for research involving maintenance of cell cultures	
	ELISA	‘	and transplant studies.	
	Western blotting	Not widely available		
	Polymerase Chain Reaction		Confirmation of serology positive result	
Measles (Paramyxovirus- Morbillivirus)	Serology	IgG assays do not detect early disease	Quarantine Screening	
	Mutiplex Fluorometric Immunoassay		Post-vaccination to determine seroconversion	Monitor clinical signs. Evaluate risks of contamination of the colony and staff. Evaluate impact on research
	ELISA			
	Polymerase Chain Reaction		Oral swabs and blood. Confirmation of infection	
				
Hepatitis Viruses A, B and C	Serology	IgG assays do not detect early disease	Quarantine Screening	Monitor biochemistry, evaluate risks of contamination to the colony and staff. Evaluate risks on research
	ELISA		Post vaccination to determine seroconversion	
			Infection confirmation in unvaccinated groups	
Rabies (Rhabdovirus, Lyssavirus)	Serology		Quarantine Screening	
	Fluoresent Antibody Virus Neutralisation	Not widely available	Post vaccination to determine seroconversion	Separate animal from non infected animals. Take action to protect staff from this severe zoonosis
	Rapid Fluorescent Focus Inhibition Test	Not widely available		
	ELISA	Does not detect neutralising antibody		
	Polymerase Chain Reaction		Confirmation of infection	
Monkeypox (Poxvirus - Orthopoxvirus)	Serology	Does not detect early disease	Quarantine Screening	Monitor clinical signs, evaluate risks of contamination of the colony and staff, Evaluate impact on research.
	ELISA			
	MFIA			
	Polymerase Chain Reaction			
Filoviruses	Serology	Does not detect early disease	Quarantine Screening	Separate snimal from non-infected animals. Take action to protect staff from this severe zoonosis.
	Polymerase Chain Reaction			
Mycobacteria	Tuberculin Skin Test	Prone to false positives and false negatives	Quarantine Screening	Separate animal from non-infected aniamsls. Take actions to protect staff from this severe zoonosis.
	Serology	Some animals may not seroconvert	Health Screening	
	Mutiplex Fluorometric ImmunoAssay	Does not detect early infection.		
	Culture	Slow growing. Culture may take up to 6 weeks. Requires tracheal washes.		
	Polymerase Chain Reaction	Difficult specimen collection requiring tracheal washes		
Enterobacteriacae:	Culture	Variable sensitivity depending on method	Quarantine Screening	Monitor clinical signs. Evaluate risks of contamination to colony and staff. Evaluate impact on research. Treatment if possible.
Salmonella species		Shedding in faeces may be intermittent	Health Screening	
Shigella species				
Yersinia species				
Campylobacter species	Culture	Shedding in faeces may be intermittent		Monitor clinical signs. Evaluate risks of contamination to staff.
Leptospira interrogans	Serology	Does not detect early infection		Monitor clinical signs. Evaluate risks of contamination to the colony and staff. Evaluate impact on research and treatment if possible.
	Microscopic Aggultination Test	Requires paired sera		
		Cross reactivity between serovars		
	ELISA	ELISA has poor specificity with high false positives.		
	Culture	Only available in specialist laboratories		
	Polymerase Chain Reaction	More sensitive than MAT but only detects positive during acute phase	Confirm serology positive results	
Klebsiella pneumoniae	Culture	Poor sensitivity unless associated with clinical infection		Monitor clinical signs. Evaluate risks to staff.
	Polymerase Chain Reaction			
				
Burkholderia pseudomallei	Culture	Poor sensitivity		Monitor clinical signs
	Polymerase Chain Reaction	Not widely available		Monitor clinical signs. Evaluate risks of contamination to the colony and staff. Evaluate impact on research
Toxoplasma gondii	Serology			
	ELISA			
	MFIA			
	Sabin -Feldman Dye Test	Gold standard - Only available in specialist reference laboratories		
	Polymerase Chain Reaction			
	Histopathology			
Intestinal Protozoa	Faecal flotation/Microscopy		Quarantine Screening Health Screening	Monitor clinical signs. Evaluate risks of contamination to the colony and staff. Evaluate impact on research and treatment if possible
	Formyl-ether concentration/Microscopy	Poor sensitivity reduced by pooling samples		
	Faecal Antigen test EIA	Variable sensitivity and specificity		
	Rapid Immunochromatographic test			
	Polymerase Chain Reaction			
	Histopathology			
Intestinal Helminths	Faecal flotation/Microscopy		Quarantine Screening	Monitor clinical signs. Evaluate risks of contamination to the colony and staff. Evaluate impact on research and treatment if possible
	Formyl-ether concentration/Microscopy		Health Screening	
	Polymerase Chain Reaction			
Pneumonyssus simicola	Histopathology	Post-mortem sampling		Monitor clinical signs. Evaluate risks of contamination to the colony. Evaluate impact on research and treatment if possible.
Ectoparasites	Microscopy		Quarantine Screening	Monitor clinical signs. Evaluate risks of contamination to the colony. Evaluate impact on research and treatment if possible.
Fungal Infection	Culture	Fungal cultures may take up to 2 weeks	Health Screening	Monitor clinical signs. Evaluate risks of contamination to the colony. Evaluate impact on research and treatment if possible

## Health-monitoring reports and certification

Standardization of health-monitoring reports and certification is important to prevent confusion. In order for a realistic risk assessment, reports and certificates should provide clarity about whether they relate only to the animals identified on the report, or if they relate to the source of those animals. Test-negative animals sourced from a colony in which the infectious agent of interest is endemic should be regarded as a higher risk than test-negative animals from a test-negative source.

The report or certificate should also provide information about the laboratory performing the testing with regard to their accreditation and quality assurance. Reports should provide information about the methods employed including published sensitivity and specificity data where possible. If reporting or certification relates to groups of animals, the numbers of animals sampled and tested relative to the population of the group, the frequency of testing and how these are selected should also be reported, in order to assess the confidence that can be placed in the conclusions drawn from the results.

All facilities that breed or house NHPs for biomedical research should implement risk-based programmes for the detection and control of potential pathogens and other agents of interest. Where appropriate these should be aimed at the eradication of the agent in question, or prevent its introduction, to ensure the health of animals and staff and also to prevent compromise to research programmes. It must be accepted, however, that the aim of eliminating all infectious agents from colonies of NHPs may not be a realistic goal nor, in fact, desirable.

## Infectious agents of importance

This paper considers the species of NHPs most commonly used in biomedical research: *Macaca mulatta* (Rhesus macaque) and *Macaca fascicularis* (Cynomolgus macaque). Additional comments relevant to *Callithrix jacchus* (Common marmoset), *Saimiri sciureus* (Squirrel monkey), *Chlorocebus aethiops* (Vervet monkey) and *Papio* sp. (Baboons) are included where species specific examples or discussion is considered necessary.

The organisms of importance discussed are limited to the major pathogenic, zoonotic and interfering agents relevant to NHPs from captive bred sources. In specific circumstances it may be important to consider additional agents depending on, for example, where the facility includes an outdoor environment, where there are particular requirements for the research being undertaken or in rare situations where wild-caught animals may be used.

## Viruses

### Cercopithecine herpesvirus 1, Herpesvirus simiae, Herpes B

Herpes B virus is arguably the most significant zoonotic infection to consider when working with macaques. Human infection is rare but frequently lethal.^7–9^ Herpes B virus has been mainly associated with Asian macaques.^10^ However, Herpes B virus has also been reported in a colony of Capuchin monkeys that developed persistent and asymptomatic Herpes B virus infection, while housed in the proximity of Rhesus macaques without direct contact. Other, sometimes fatal, herpes virus infections have been documented in various non-human primate species including *Cebus apella*, *Papio* sp. and in *Callithrix jacchus*.^11,12^

In macaques, infection is usually transmitted via bites, scratches, sexual contact or contact between saliva and other bodily fluids from an infected animal with defects of the mucous membranes or skin. Vertical transmission can also occur. Viremia is intermittent and does not always coincide with the presence of clinical signs of infection. Clinical signs are predominantly vesicular lesions along the muco-cutaneous junctions and in the oral cavity. Peak seroconversion usually occurs between two and three years of age during adolescence and during the onset of sexual maturity.

Like other herpes viruses, Herpes B virus exhibits latency, particularly within the trigeminal ganglion. This leads to lifelong infection which may remain quiescent for considerable periods. During these periods, antibody titres to the initial infection may decrease to a level below the detection limits of serological assays. Titres may rise again periodically, particularly at times of ‘stress’ or where there is a possibility of immunosuppression. This suggests that a single negative antibody test result, particularly when only an individual animal is being tested, cannot be relied upon to confirm the absence of infection. Absence of this organism is more reliably detected in group situations following serial testing. However, it should be noted that some animals have developed serological evidence of infection many years after the establishment and maintenance of a closed, entirely seronegative colony. PCR carried out on swabs collected from the buccal cavity may provide additional useful information. A positive result can only be obtained if the animal is viraemic and the virus is being shed at the time of sampling. Direct detection methods such as PCR can be useful to determine the risk of Herpes B virus infection associated with potential exposure to animal handlers, for example after bites or other potential exposure.

### Immunosuppressive retroviruses

Retroviruses are RNA viruses which are characterized by the presence of the enzyme, reverse transcriptase. Reverse transcriptase converts single stranded RNA into double stranded cDNA. In retroviruses, cDNA is integrated into the host-cell genome to make new RNA copies or it remains latent for a period which may be measured in years. During this period latently-infected animals can frequently be antibody negative, therefore sensitivity of this type of testing is limited and a negative serological result does not confirm the absence of infection. Direct methods such as PCR to detect the viral genome is more useful in this regard.

Although infections with these viruses appear to have minor or absent clinical signs in the animals they infect, the immunosuppression associated with them increases the morbidity and mortality associated with other pathogens. These organisms can also have significant adverse effects on immunological research, vaccine efficacy or research involving therapies against other retroviruses

#### Simian Immunodeficiency Virus (SIV)

SIV is common amongst populations of African monkeys including *Chlorocebus aethiops* and *Papio* spp. which generally remain as asymptomatic carriers of the infection. SIV is not naturally present in wild populations of Asian macaques. However, these animals can be readily infected with SIV making SIV a useful model of HIV for researchers in this field. The risk of transmission of SIV in macaque colonies other than those involved in HIV research is considered low. Ongoing health screening for this pathogen is considered unnecessary except when animals are being sourced for addition to the colony.

SIV carries the potential for zoonotic infection. However, very few cases of seroconversion in people exposed to this virus have been reported and pathogenicity in humans has yet to be demonstrated.

#### Simian T-cell Lymphotrophic Virus (STLV)

STLV, a c-type member of the oncornavirus subgroup of retroviruses is endemic in Old World NHPs. Three serotypes have been reported with STLV-A being by far the most prevalent, particularly in macaques, but more than 18 different species of Old World NHPs including baboons and African green monkeys have been documented with STLV infections.^13^

STLV is highly T-cell specific. Transmission is primarily by the transfer of semen or cervical secretions during breeding. STLV can also be transmitted via breast milk to infants. Prevalence increases as animals age and become sexually mature.

Seroconversion is slow, often taking as long as six to nine months even in immunocompetent animals. In individuals co-infected with another immunosuppressive retrovirus this may be extended to several years. Although there are reports of aetiological links between STLV infection and malignant lymphomas and lymphoproliferative disease in some primate species, most infected animals remain apparently healthy and asymptomatic albeit with possible deleterious effects on the immune system.

STLV-1 shares between 90 and 95% genetic homology with Human T-cell Lymphotrophic Virus-1 (HTLV-1) (together referred to as PTLVs – Primate T-cell Lymphotrophic Viruses). In humans, HTLV-1 is considered the aetiological agent of adult T-cell leukaemia, lymphoma and the progressive neurological disease Spastic Paresis. Although little has been reported, this homology indicates that there may be potential for STLV-1 to establish infections in man.

#### Simian type D Retroviruses (SRV(D))

The SRV(D) are a group of closely-related viruses which have been isolated from many species of Asian NHPs including macaques. Different serotypes predominate in the different NHP species. Infection can result in a wide range of clinical manifestations from a subclinical carrier state to rapidly fatal immunosuppressive disease. Direct animal-to-animal contact is the most common route of transmission.

In infected animals SRV(D) can often be demonstrated in a variety of tissues and organs. However, a significant proportion (30%) of infected macaques may remain seronegative when tested for SRV(D) specific antibodies. This must be considered when designing health-screening protocols.

#### Simian Foamy Viruses (SFV (Spumavirus))

Foamy viruses are highly prevalent in virtually all species of NHPs, approaching 100% prevalence in many populations. They appear to be non-pathogenic but can interfere with studies requiring the growth or maintenance of cell cultures and transplant studies because of their highly cytolytic effects.

### Measles (Paramyxovirus: Morbillivirus)

Measles is a highly infectious paramyxovirus of the genus *Morbillivirus*. Humans are the natural host for this virus, but most non-human primate species, particularly macaques, are susceptible to infection. Infection with measles can have serious consequences including fatality.^14^ Measles-virus infections in marmosets can rapidly spread through a colony resulting in high morbidity and mortality.^15^

Measles infections in colonies of NHP are generally the result of transmission from animal-care staff. Transmission occurs via aerosols, although direct contact and fomite transmission may also be possible. Clinical signs in NHP generally appear approximately a week following infection and are similar to those seen in man. The predominant signs being a maculopapular skin rash, conjunctivitis, blepharitis and malaise. Measles is immunosuppressive causing transient humoral and cell-mediated immune dysfunction.

The value of health screening colonies of NHP with respect to measles is debatable. The clinical signs of disease are obvious and positive antibody-test results confirm previous exposure (or vaccination). Alternatively, the possible benefits of introducing a vaccination policy to protect the animals and confirming the animal-care staff are vaccinated, should be considered.

### Hepatitis (Orthohepadnavirus and Picornavirus)

Historically, Hepatitis viruses including Hepatitis A and Hepatitis B, have been considered an infection of humans and the great apes. However, it should be borne in mind that there is now one report which demonstrates that naturally occurring transmissible chronic Hepatitis B virus infections can exist among some wild *Macaca fascicularis* populations.^16^ In some countries, Hepatitis B vaccination is routinely carried out on captive NHPs and may be a requirement.

A high seroprevalence to simian Hepatitis A, which may carry the possibility of zoonotic infection, has been reported in non-human primate colonies.^17^ Infection is usually via the faecal–oral route, is self-limiting and asymptomatic. Control usually centres on maintaining appropriate environmental hygiene and the use of personal protective equipment by those in contact with the animals or their environment.

### Rabies (Rhabdovirus, Lyssavirus)

In closed colonies of captive-bred NHPs, rabies is not generally of concern. However, a route of transmission is plausible where animals have access to outdoor pens allowing the possibility of interaction with local wildlife.

In some European countries serological testing and/or vaccination against this zoonotic agent is required for regulatory reasons before animals can be imported from countries or regions where rabies remains endemic.

### Monkeypox (Poxvirus: Orthopoxvirus)

Cases of monkeypox, which is closely related to smallpox in man, have been reported in animals from the African continent.^18^ With the exception of animals sourced from areas where infection is endemic, it is not considered necessary to screen for this agent.

### Filoviruses

Historically, cases of infection with Marburg virus and strains of Ebola have been reported within captive colonies of NHPs. Screening for this agent is not considered necessary except in circumstances when animals are sourced from locations where infection is endemic (e.g. Africa) and should be performed during quarantine screening.

New World NHPs, particularly *Callithrix jacchus*, are well known to have a greater susceptibility to many anthroponotic viral infections than many of the Old World NHPs although there is little evidence for natural transmission of many of these viruses within primate colonies. Outbreaks of Parainfluenza 1 (Sendai-like) virus have, for example, been reported in Common marmosets with a relatively high morbidity but low mortality.^19^

## Bacteria

### Mycobacteria

Opportunistic infection with various ‘atypical’ Mycobacterial species including *M. avium* and *M. intracellulare*, are frequently diagnosed, particularly in immunocompromised non-human primate species. However, the Mycobacterial species of greatest concern, are *M. tuberculosis* and *M. bovis* due to their zoonotic and anthroponotic potential.^20,21^ Although largely controlled in captive-bred animals in Europe, these agents remain a significant threat in animals imported from other regions of the world where there are high rates of human infection or where there is potential for the primates to have contact with endemically infected wildlife.

Mycobacterial infections are characterized by a prolonged incubation period with respiratory disease usually becoming the most common clinical manifestation of infection. Systemic dissemination to almost any organ can occur, as well as latency. Treatment is prolonged and is not recommended.

Regular health screening of animals and personnel is essential. The frequency of health screening should be determined according to risk and should be carried out on at least an annual basis for all captive NHPs. Screening is commonly based on the tuberculin skin test (TST). In NHPs, the test is performed by injecting either Mammalian Old Tuberculin (MOT) or *M. bovis* Purified Protein Derivative (PPD) intra-dermally into the skin of the eyelid close to its margin, or the hair-free skin of the abdomen or both. The injection site is monitored at 24, 48 and 72 hours for the development of a delayed hypersensitivity response with the degree of erythema and oedema being categorized on an increasing scale. MOT is generally considered the test reagent of choice due to its enhanced ability to evoke an immune response providing improved test sensitivity. However, this can also result in an unacceptably high proportion of false positive results. PPD may be used when MOT availability in Europe is limited. Consideration must be given to the possibility of false negative results which may occur both very early during the onset of infection or in the advanced stages of infection, if excessive testing leads to anergy or where there is concomitant infection with an immunosuppressive agent.

Given the complexity in diagnosing Mycobacterial infection, all positive TST results should be investigated further to confirm health status. Confirmatory tests which could be considered include:

- Thoracic radiography, but pulmonary lesions are not specific for tuberculosis and may not be apparent very early in the course of the disease.
- Serological or γ-interferon testing using blood or serum samples.^22–24^
- Culture or PCR testing for *M. tuberculosis* or *M. bovis* which can be carried out on sputum, broncho-alveolar lavage or necropsy samples.

Although *Callithrix jacchus* is very susceptible to experimentally induced infections with *M. tuberculosis*^25^ natural Mycobacterial infections in this species appear rare with infrequent reports in the literature.

### Enterobacteriaceae

NHP are frequently asymptomatic carriers of Enterobacteriacae. Enterobacteriacae have the potential to cause disease, particularly in immunocompromised animals, and they also have the potential for zoonotic spread. The bacterial species likely to be of greatest interest include *Salmonella* sp., *Shigella* sp., *Campylobacter* sp. and *Yersinia* sp.^26–28^ Pest control policies for insects, rodents, birds and other wildlife are important biosecurity measures in preventing the transmission of these organisms to NHP colonies. Biosecurity measures should also be in place to prevent anthroponotic transmission.

*Salmonellae*, including species such as *S. typhimurium* and *S. enteritidis* have the potential to cause generalized systemic illness as well as gastro-intestinal disease. In such situations treatment may be warranted. However, in less severe situations this may prolong faecal shedding of the organism or even induce a carrier status. Salmonellosis is a reportable disease in many European countries.

Shigellosis is, in many respects similar to Salmonellosis in NHPs with *S. flexneri* being the most common species isolated.

*Yersinia enterocolitica* and *Y. pseudotuberculosis* have both been associated with disease in colonies of NHPs (*Y. pestis* is not generally considered a risk except possibly in areas where this is endemic in the local rat population). *Y. pseudotuberculosis* in particular has been associated with severe infections with systemic as well as gastro-intestinal manifestations (including reproductive failure) and high mortality amongst groups of marmosets. Vaccination in such situations should be considered.

Screening for the various potential Enterobacterial pathogens generally relies on microbiological culture. Detecting these organisms particularly when screening apparently healthy populations, can lead to false negative results. This is due to limitations of the currently available methods and intermittent or low-level excretion of these organisms. Detection of Enterobacteriacae can be improved by careful sample selection (faecal samples are generally regarded as preferable to rectal swabs), sampling strategy (collecting a rectal swab for culture daily on three consecutive days) will vastly increase sensitivity compared to collecting a single rectal swab for culture, especially when there is intermittent excretion of the target organism. The use of specific enrichment techniques such as Selenite F broth and Chromogenic agars will also improve the sensitivity of culture. PCR can also be useful to confirm the identity of suspect organisms following culture but may be considered overly sensitive for use directly on faecal samples.

Before embarking on any screening programme for potentially pathogenic Enterobacteria, consideration should be given to any action that may need to be taken should one of the target bacterial species be isolated. This is particularly true if the samples are from animals where clinical signs are absent. Treatment may be considered if prevalence is high, however, although this may prevent or reduce the severity of disease or reduce bacterial shedding, it may also prolong bacterial shedding and induce a carrier state and is unlikely to ever eliminate infection. If treatment is considered necessary, an antibiotic sensitivity test should be performed to choose an effective antibiotic. A careful harm-benefit analysis should be performed when treating a whole population to avoid the development of antibiotic resistance.

### Campylobacteraceae

Many Campylobacter species can be isolated from the faeces of clinically healthy and diseased NHPs. The significance of Campylobacters is debatable and may depend on the bacterial species/strain and the presenting clinical signs. Treatment is not usually recommended unless the clinical signs are severe.

### Leptospira interrogans

Various Leptospiral species can infect NHPs and cause disease, with zoonotic potential, and even death. Screening is recommended when risk is considered highest, in facilities where contact with infected wild rodents and other wildlife cannot be prevented.

### Klebsiella pneumoniae

*K. pneumoniae* is associated with severe infections with high mortality in marmoset colonies.^29^ Infections more often present at three months of age when maternal antibodies decrease and the individual antibody production of the infant is not yet effective.

### Burkholderia (formerly Pseudomonas) pseudomallei

*B. pseudomallei* is an environmental saprophyte found in sub-tropical and tropical regions of mainly South-East Asia and Australia. In these endemic areas it can easily be isolated from samples of soil and water. Infection resulting in the disease ‘melioidosis’ (mainly cutaneous lesions) occurs via aerosol transmission or ingestion. Treatment is generally not effective.

Only animals originating from areas in which this disease is endemic are considered at risk and routine health screening is not required in European colonies.

## Protozoal and parasitic infections

### Toxoplasma gondii

Toxoplasma is only considered a risk where there is access by primates to outdoor enclosures allowing possible contact with infected cats. Clinical disease is more severe in new world primates than old world primates and primary infection may be fatal. Toxoplasma has zoonotic potential.

### Intestinal protozoal parasites

This includes *Entamoeba histolytica* (*E. coli* and *E. dispar* are considered non-pathogenic), *Balantidium coli* and *Giardia* sp. In low numbers these organisms can be considered part of the normal gut flora, causing no adverse effect but in higher numbers, particularly in immunocompromised individuals intermittent to severe diarrhoea or dysentery may be seen.^30,31^ Intestinal protozoal parasites of NHPs carry the risk of zoonotic infection.

In apparently healthy animals treatment is not usually required or recommended but in debilitated animals treatment with metronidazole can be effective.

*Cryptosporidium parvum* infections can occasionally occur in facilities where NHPs have access outside enclosures where puddles of standing water may persist. This agent has zoonotic potential.

Health screening for the presence of intestinal protozoal parasites is generally carried out on pooled faecal samples using flotation, formyl-ether concentration and microscopy examination although PCR testing is available. In most cases, antibiotic treatments are available.

### Intestinal helminth parasitism

Parasitism involving various helminth species including *Strongyloides* and *Trichuris* can affect various species of NHP. However, it is rare in animals internally housed and fed pelleted diets supplemented with washed fruit and vegetables. When infection is detected, usually by microscopic examination of pooled faecal samples for the detection of parasite eggs, it is usually without clinical signs. Various anthelmintic treatment options are available.

Special consideration should be given to *Echinococcus multilocularis*.^32^ Echinococcus is only considered a risk if primates have access to outdoor enclosures, allowing possible contact with infected foxes in areas where this parasite is endemic. However, Echinococcosis can occur after a long period of time after importation from an endemic area; this might include several changes of facilities until the illness finally develops. Serological and PCR screening is possible but postmortem examination provides the most useful information.

### Pneumonyssus simicola

Lung mites are not considered a significant problem in European captive NHPs although they can be seen in macaques imported from regions of the world where they are endemic.^33^ Coughing is a common clinical sign of Pneumonyssus infection. Diagnosis is usually made at postmortem and often coincidental. Treatment with effective endectocides is available.

### Ectoparasites

As in other mammals, mites and lice are a theoretical risk in NHP. Especially in outdoor facilities, an ectoparasiticide programme should be implemented.

### Fungal infections

Dermatophytosis, usually caused by *Trichophyton* sp., resulting in areas of roughly circular alopecia with cutaneous inflammation around the border will normally be detected during routine clinical examination. The diagnosis can be made by collecting skin scrapings for fungal culture or microscopic examination. *Trichophyton* sp. are zoonotic and humans may become infected by direct contact.

## Conclusion

It is important to consider that all NHP facilities have their own specific requirements and that these recommendations should be adapted to suit local needs. These requirements will depend on the geographical location, regulatory requirements, prevalent endemic organisms and the research undertaken.

This review is not exhaustive and continuous efforts to increase the knowledge of infectious diseases of NHPs should be undertaken. Regular monitoring of scientific developments in this field is required for the optimal management of NHP colonies. We encourage other authors to update this guidance as necessary.
